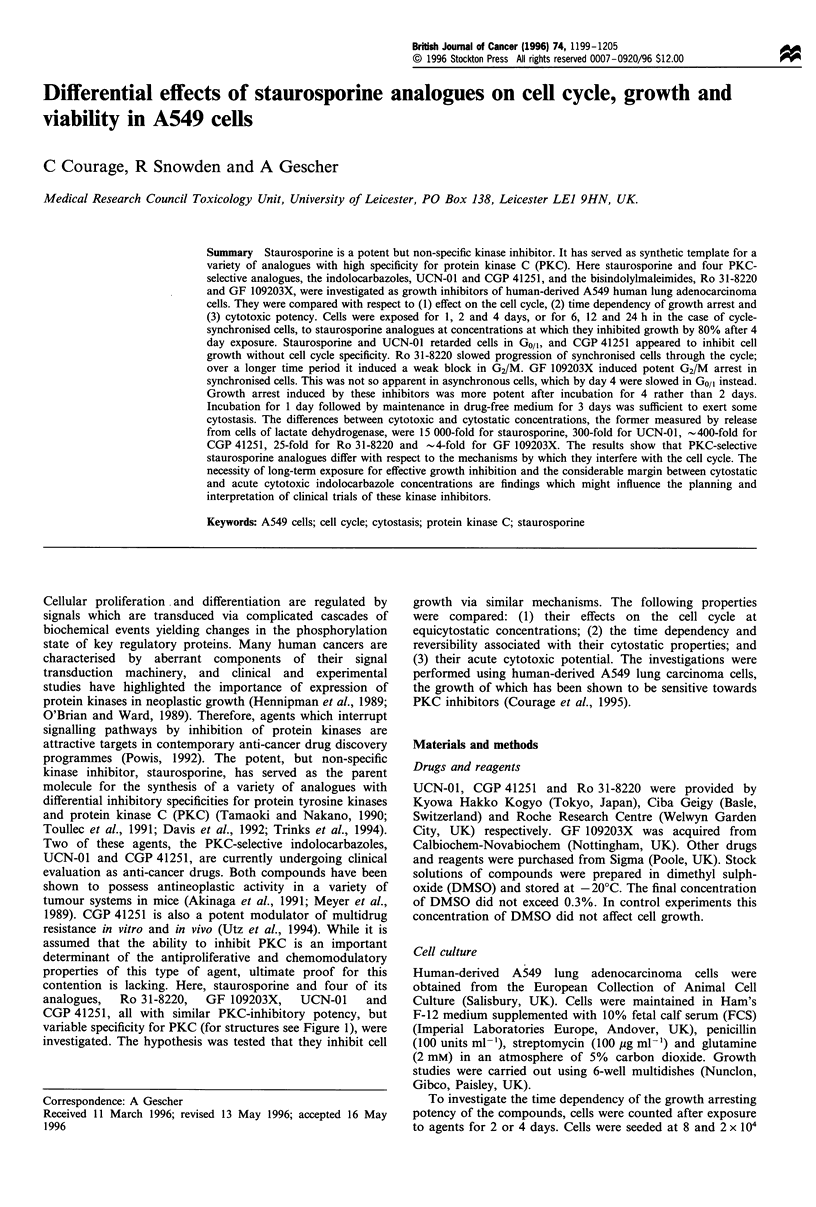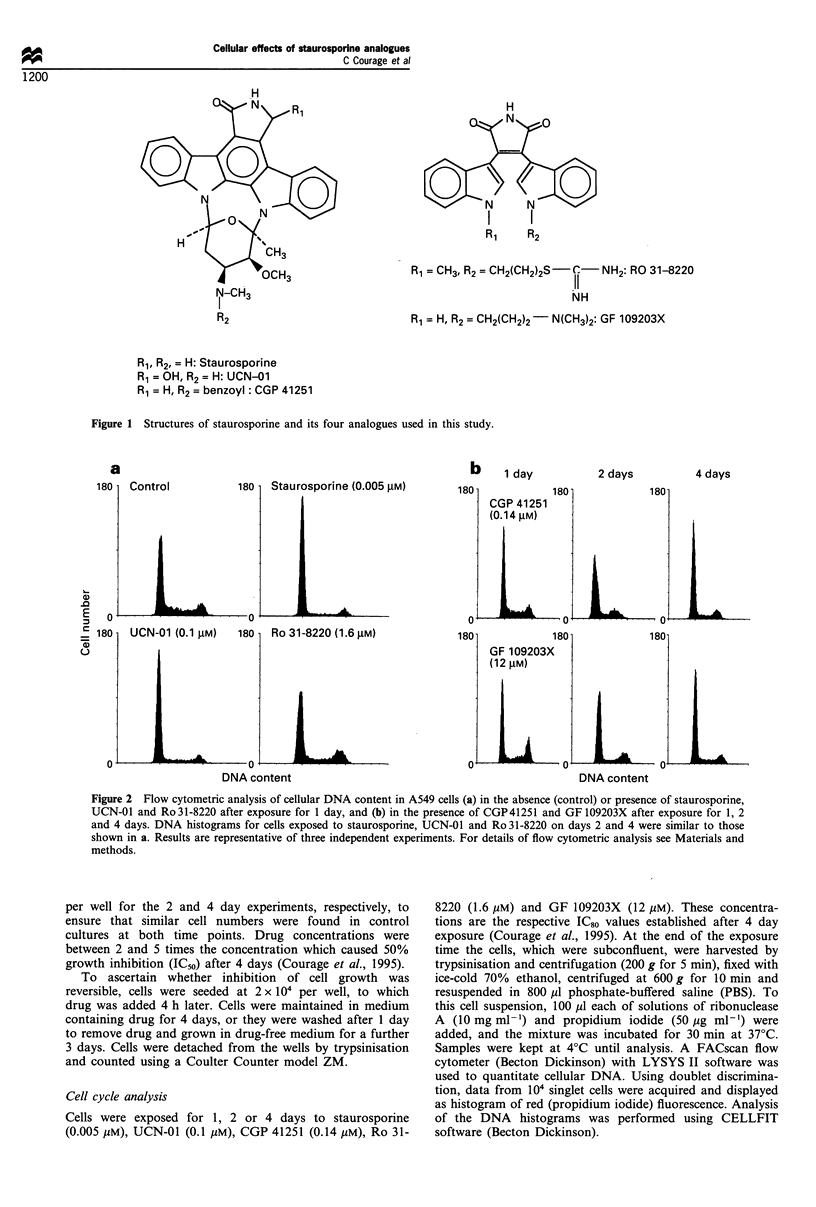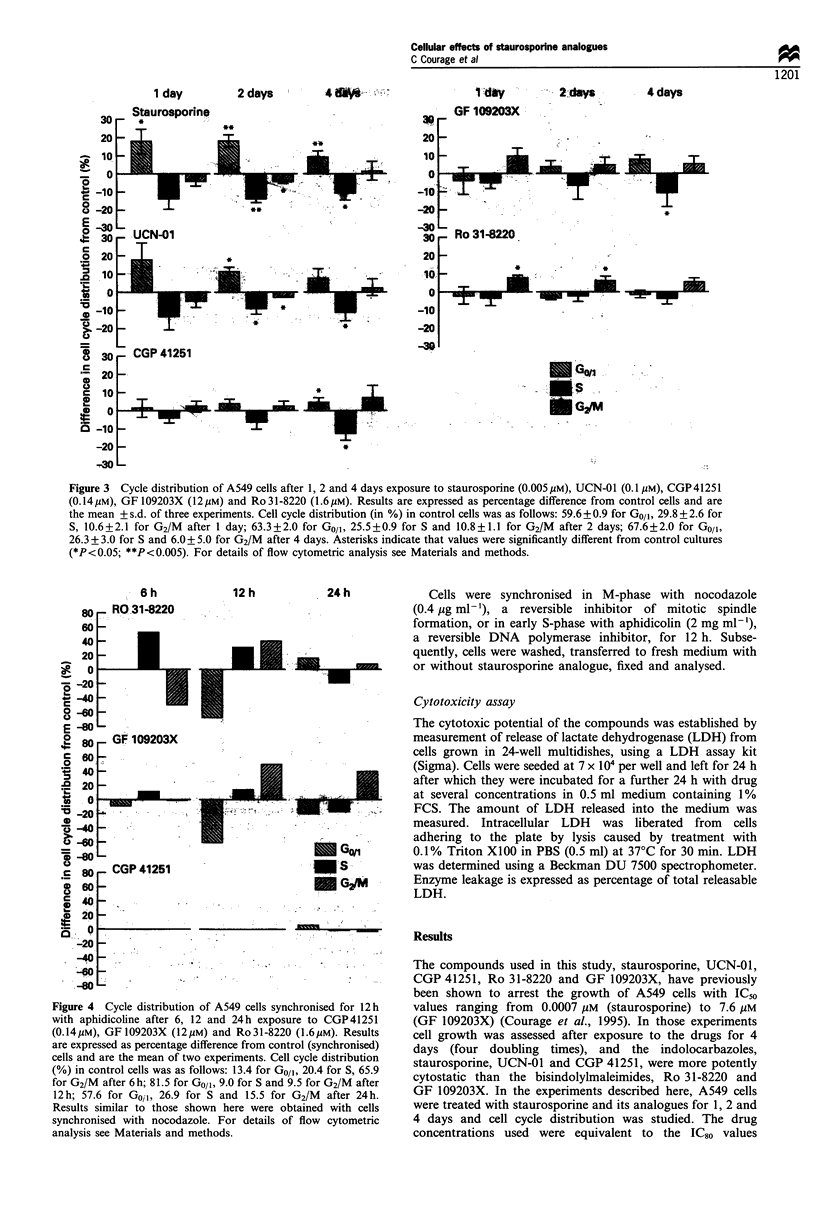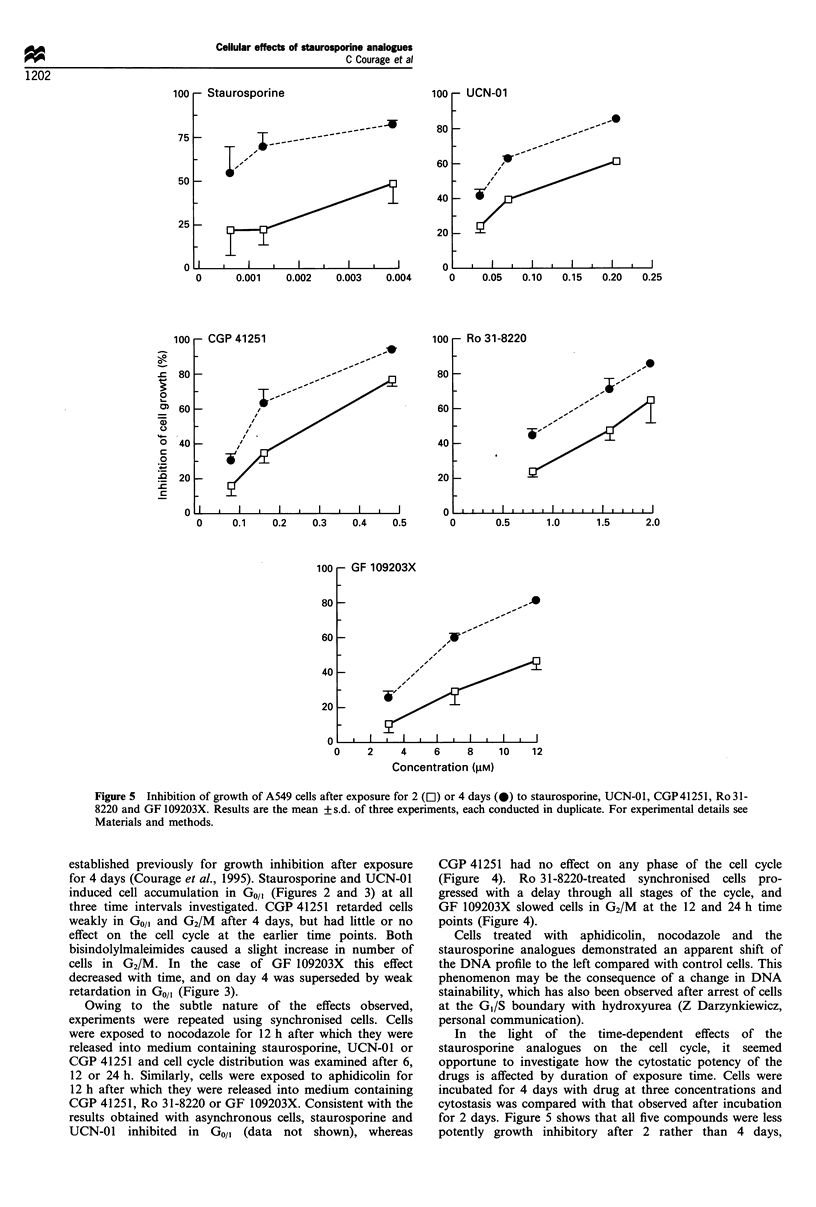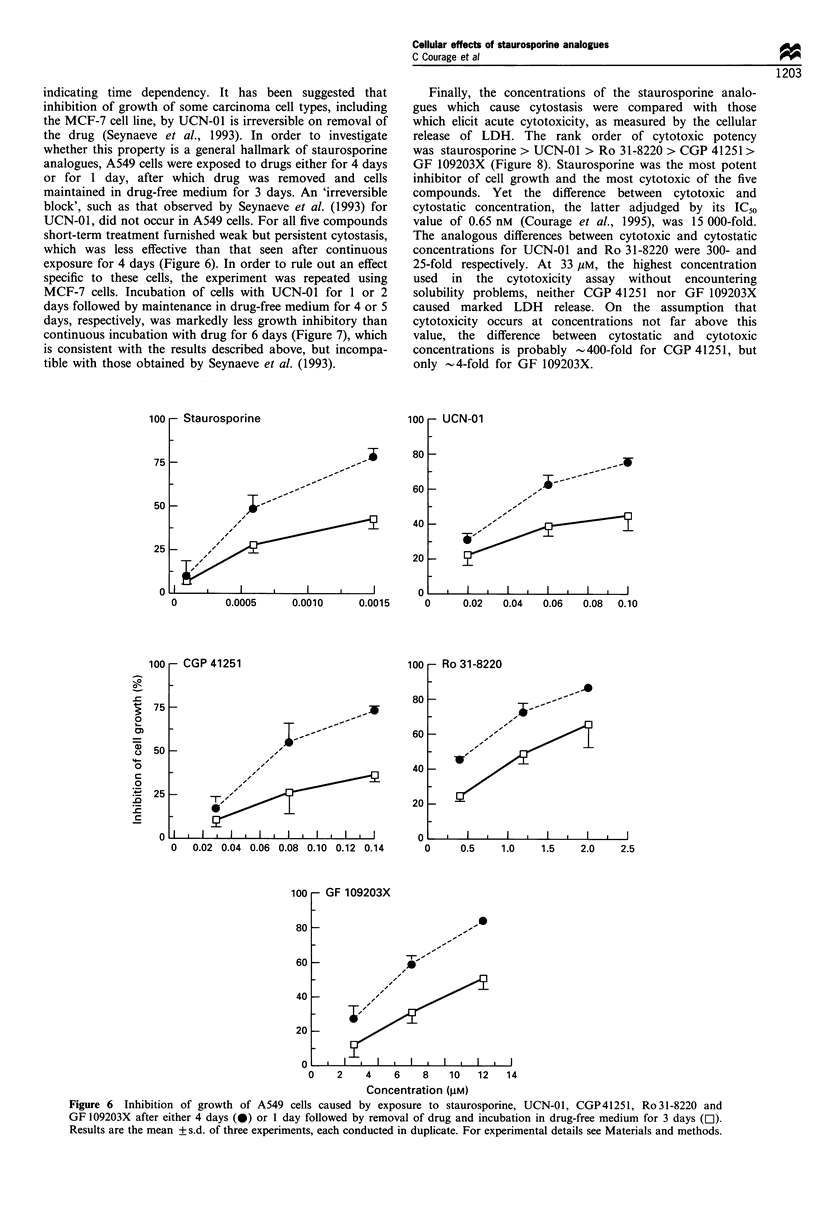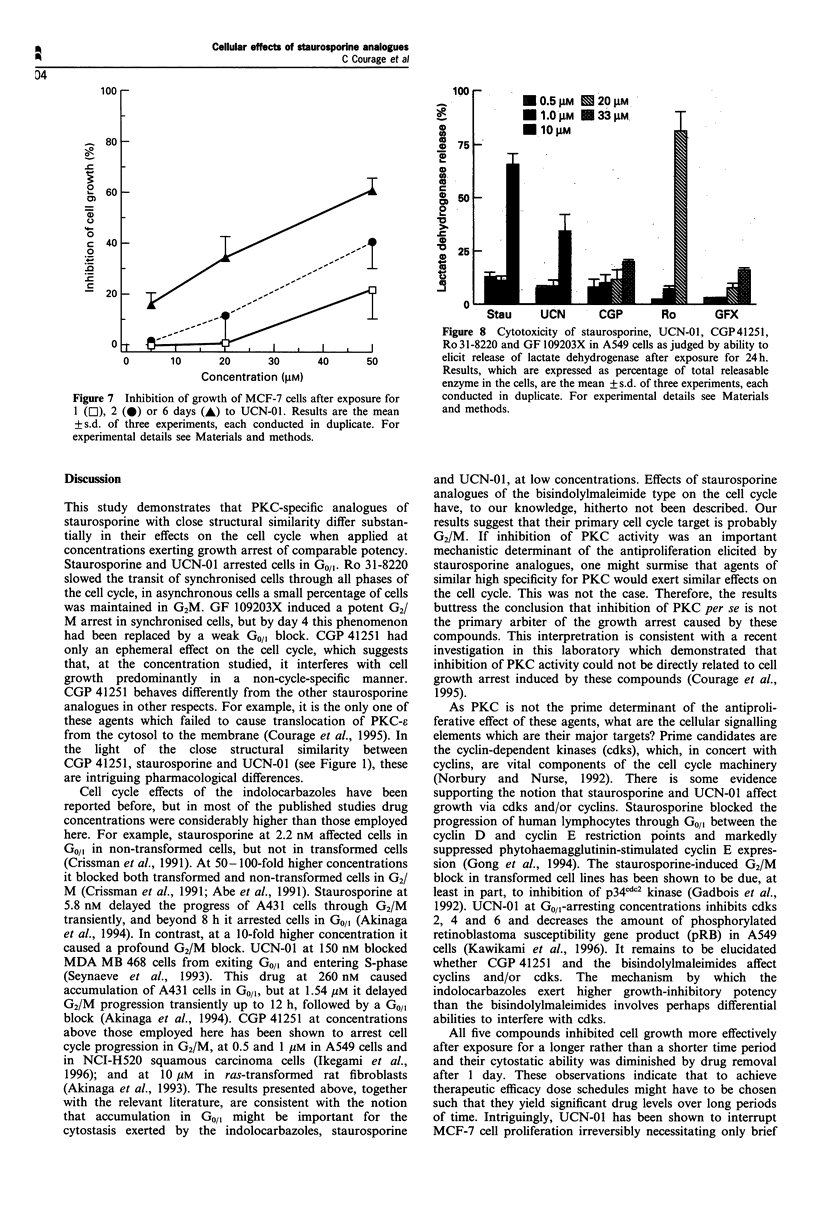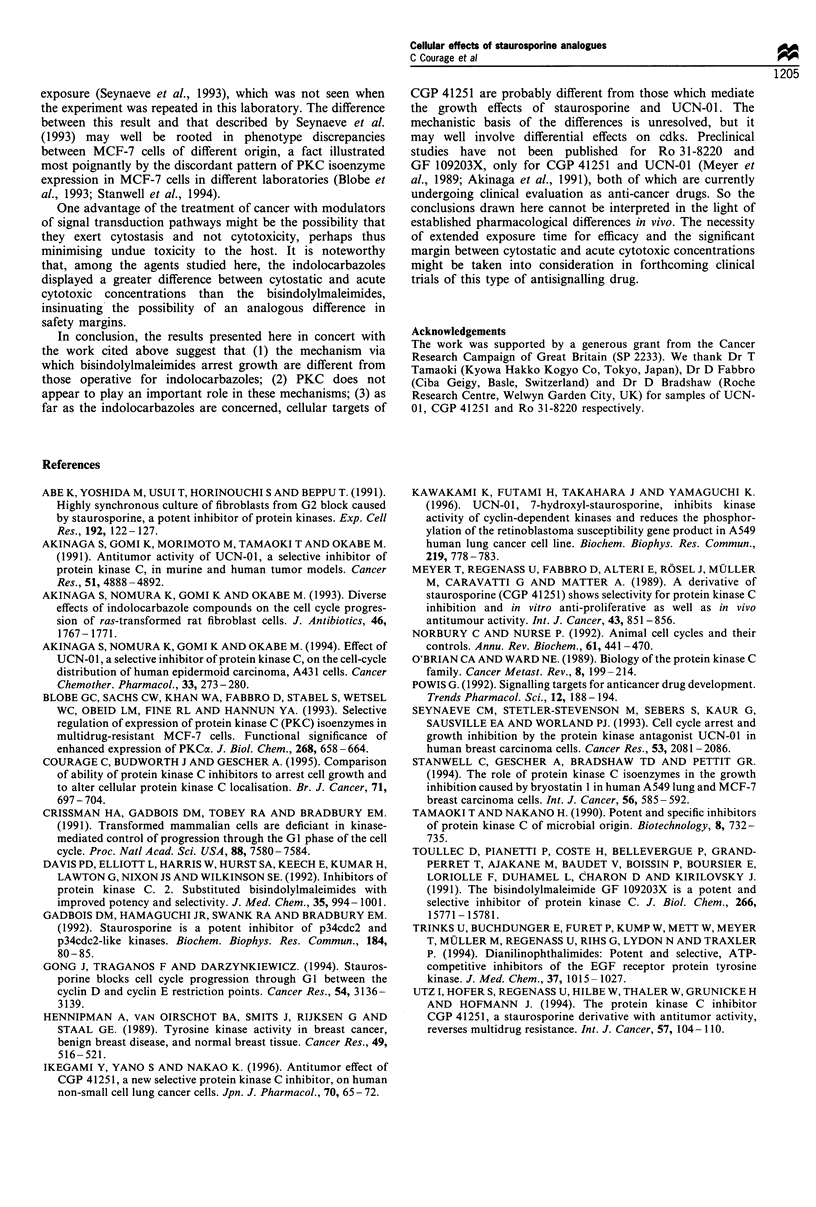# Differential effects of staurosporine analogues on cell cycle, growth and viability in A549 cells.

**DOI:** 10.1038/bjc.1996.517

**Published:** 1996-10

**Authors:** C. Courage, R. Snowden, A. Gescher

**Affiliations:** Medical Research Council Toxicology Unit, University of Leicester, UK.

## Abstract

Staurosporine is a potent but non-specific kinase inhibitor. It has served as synthetic template for a variety of analogues, the indolocarbazoles, UCN-01 and CGP 41251, and the bisindolylmaleimides, Ro 31-8220 and GF 109203X, were investigated as growth inhibitors of human-derived A549 human lung adenocarcinoma cells. They were compared with respect to (1) effect on the cell cycle, (2) time dependency of growth arrest and (3) cytotoxic potency. Cells were exposed for 1, 2 and 4 days, or for 6, 12 and 24 h in the case of cycle-synchronised cells, to staurosporine analogues at concentrations at which they inhibited growth by 80% after 4 day exposure. Staurosporine and UCN-01 retarded cells in G0/1, and CGP 41251 appeared to inhibit cell growth without cell cycle specificity. Ro 31-8220 slowed progression of synchronised cells through the cycle; over a longer time period it induced a weak block in G2/M. GF 109203X induced potent G2/M arrest in synchronised cells. This was not so apparent in asynchronous cells, which by day 4 were slowed in G0/1 instead. Growth arrest induced by these inhibitors was more potent after incubation for 4 rather than 2 days. Incubation for 1 day followed by maintenance in drug-free medium for 3 days was sufficient to exert some cytostasis. The differences between cytotoxic and cytostatic concentrations, the former measured by release from cells of lactate dehydrogenase, were 15 000-fold for staurosporine, 300-fold for UCN-01, approximately 400-fold for CGP 41251, 25-fold for Ro 31-8220 and approximately 4-fold for GF 109203X. The results show that PKC-selective staurosporine analogues differ with respect to the mechanisms by which they interfere with the cell cycle. The necessity of long-term exposure for effective growth inhibition and the considerable margin between cytostatic and acute cytotoxic indolocarbazole concentrations are findings which might influence the planning and interpretation of clinical trials of these kinase inhibitors.


British Journal of Cancer (1996) 74, 1199-1205
© 1996 Stockton Press All rights reserved 0007-0920/96 $12.00

### Differential effects of staurosporine analogues on cell cycle, growth and viability in A549 cells

C Courage, R Snowden and A Gescher

*Medical Research Council Toxicology Unit, University of Leicester, PO Box 138, Leicester LE1 9HN, UK.*

**Summary** Staurosporine is a potent but non-specific kinase inhibitor. It has served as synthetic template for a variety of analogues with high specificity for protein kinase C (PKC). Here staurosporine and four PKC-selective analogues, the indolocarbazoles, UCN-01 and CGP 41251, and the bisindolylmaleimides, Ro 31-8220 and GF 109203X, were investigated as growth inhibitors of human-derived A549 human lung adenocarcinoma cells. They were compared with respect to (1) effect on the cell cycle, (2) time dependency of growth arrest and (3) cytotoxic potency. Cells were exposed for 1, 2 and 4 days, or for 6, 12 and 24 h in the case of cycle-synchronised cells, to staurosporine analogues at concentrations at which they inhibited growth by 80% after 4 day exposure. Staurosporine and UCN-01 retarded cells in $G_{0/1}$, and CGP 41251 appeared to inhibit cell growth without cell cycle specificity. Ro 31-8220 slowed progression of synchronised cells through the cycle; over a longer time period it induced a weak block in $G_2$/M. GF 109203X induced potent $G_2$/M arrest in synchronised cells. This was not so apparent in asynchronous cells, which by day 4 were slowed in $G_{0/1}$ instead. Growth arrest induced by these inhibitors was more potent after incubation for 4 rather than 2 days. Incubation for 1 day followed by maintenance in drug-free medium for 3 days was sufficient to exert some cytostasis. The differences between cytotoxic and cytostatic concentrations, the former measured by release from cells of lactate dehydrogenase, were 15 000-fold for staurosporine, 300-fold for UCN-01, ~400-fold for CGP 41251, 25-fold for Ro 31-8220 and ~4-fold for GF 109203X. The results show that PKC-selective staurosporine analogues differ with respect to the mechanisms by which they interfere with the cell cycle. The necessity of long-term exposure for effective growth inhibition and the considerable margin between cytostatic and acute cytotoxic indolocarbazole concentrations are findings which might influence the planning and interpretation of clinical trials of these kinase inhibitors.

**Keywords:** A549 cells; cell cycle; cytostasis; protein kinase C; staurosporine

Cellular proliferation and differentiation are regulated by signals which are transduced via complicated cascades of biochemical events yielding changes in the phosphorylation state of key regulatory proteins. Many human cancers are characterised by aberrant components of their signal transduction machinery, and clinical and experimental studies have highlighted the importance of expression of protein kinases in neoplastic growth (Hennipman *et al.*, 1989; O'Brian and Ward, 1989). Therefore, agents which interrupt signalling pathways by inhibition of protein kinases are attractive targets in contemporary anti-cancer drug discovery programmes (Powis, 1992). The potent, but non-specific kinase inhibitor, staurosporine, has served as the parent molecule for the synthesis of a variety of analogues with differential inhibitory specificities for protein tyrosine kinases and protein kinase C (PKC) (Tamaoki and Nakano, 1990; Toullec *et al.*, 1991; Davis *et al.*, 1992; Trinks *et al.*, 1994). Two of these agents, the PKC-selective indolocarbazoles, UCN-01 and CGP 41251, are currently undergoing clinical evaluation as anti-cancer drugs. Both compounds have been shown to possess antineoplastic activity in a variety of tumour systems in mice (Akinaga *et al.*, 1991; Meyer *et al.*, 1989). CGP 41251 is also a potent modulator of multidrug resistance *in vitro* and *in vivo* (Utz *et al.*, 1994). While it is assumed that the ability to inhibit PKC is an important determinant of the antiproliferative and chemomodulatory properties of this type of agent, ultimate proof for this contention is lacking. Here, staurosporine and four of its analogues, Ro 31-8220, GF 109203X, UCN-01 and CGP 41251, all with similar PKC-inhibitory potency, but variable specificity for PKC (for structures see Figure 1), were investigated. The hypothesis was tested that they inhibit cell growth via similar mechanisms. The following properties were compared: (1) their effects on the cell cycle at equicytostatic concentrations; (2) the time dependency and reversibility associated with their cytostatic properties; and (3) their acute cytotoxic potential. The investigations were performed using human-derived A549 lung carcinoma cells, the growth of which has been shown to be sensitive towards PKC inhibitors (Courage *et al.*, 1995).

Correspondence: A Gescher
Received 11 March 1996; revised 13 May 1996; accepted 16 May 1996

### Materials and methods

### *Drugs and reagents*

UCN-01, CGP 41251 and Ro 31-8220 were provided by Kyowa Hakko Kogyo (Tokyo, Japan), Ciba Geigy (Basle, Switzerland) and Roche Research Centre (Welwyn Garden City, UK) respectively. GF 109203X was acquired from Calbiochem-Novabiochem (Nottingham, UK). Other drugs and reagents were purchased from Sigma (Poole, UK). Stock solutions of compounds were prepared in dimethyl sulphoxide (DMSO) and stored at −20°C. The final concentration of DMSO did not exceed 0.3%. In control experiments this concentration of DMSO did not affect cell growth.

### *Cell culture*

Human-derived A549 lung adenocarcinoma cells were obtained from the European Collection of Animal Cell Culture (Salisbury, UK). Cells were maintained in Ham's F-12 medium supplemented with 10% fetal calf serum (FCS) (Imperial Laboratories Europe, Andover, UK), penicillin (100 units $ml^{-1}$), streptomycin (100 $\mu g$ $ml^{-1}$) and glutamine (2 mM) in an atmosphere of 5% carbon dioxide. Growth studies were carried out using 6-well multidishes (Nunclon, Gibco, Paisley, UK).

To investigate the time dependency of the growth arresting potency of the compounds, cells were counted after exposure to agents for 2 or 4 days. Cells were seeded at 8 and $2 \times 10^4$

**Figure 1** Structures of staurosporine and its four analogues used in this study.

**Figure 2** Flow cytometric analysis of cellular DNA content in A549 cells (**a**) in the absence (control) or presence of staurosporine, UCN-01 and Ro 31-8220 after exposure for 1 day, and (**b**) in the presence of CGP 41251 and GF 109203X after exposure for 1, 2 and 4 days. DNA histograms for cells exposed to staurosporine, UCN-01 and Ro 31-8220 on days 2 and 4 were similar to those shown in **a**. Results are representative of three independent experiments. For details of flow cytometric analysis see Materials and methods.

per well for the 2 and 4 day experiments, respectively, to ensure that similar cell numbers were found in control cultures at both time points. Drug concentrations were between 2 and 5 times the concentration which caused 50% growth inhibition ($IC_{50}$) after 4 days (Courage *et al.*, 1995).

To ascertain whether inhibition of cell growth was reversible, cells were seeded at $2\times10^4$ per well, to which drug was added 4 h later. Cells were maintained in medium containing drug for 4 days, or they were washed after 1 day to remove drug and grown in drug-free medium for a further 3 days. Cells were detached from the wells by trypsinisation and counted using a Coulter Counter model ZM.

*Cell cycle analysis*

Cells were exposed for 1, 2 or 4 days to staurosporine (0.005 $\mu$M), UCN-01 (0.1 $\mu$M), CGP 41251 (0.14 $\mu$M), Ro 31-8220 (1.6 $\mu$M) and GF 109203X (12 $\mu$M). These concentrations are the respective $IC_{80}$ values established after 4 day exposure (Courage *et al.*, 1995). At the end of the exposure time the cells, which were subconfluent, were harvested by trypsinisation and centrifugation (200 *g* for 5 min), fixed with ice-cold 70% ethanol, centrifuged at 600 *g* for 10 min and resuspended in 800 $\mu$l phosphate-buffered saline (PBS). To this cell suspension, 100 $\mu$l each of solutions of ribonuclease A (10 mg $ml^{-1}$) and propidium iodide (50 $\mu$g $ml^{-1}$) were added, and the mixture was incubated for 30 min at 37°C. Samples were kept at 4°C until analysis. A FACscan flow cytometer (Becton Dickinson) with LYSYS II software was used to quantitate cellular DNA. Using doublet discrimination, data from $10^4$ singlet cells were acquired and displayed as histogram of red (propidium iodide) fluorescence. Analysis of the DNA histograms was performed using CELLFIT software (Becton Dickinson).

**Figure 3** Cycle distribution of A549 cells after 1, 2 and 4 days exposure to staurosporine (0.005 $\mu$M), UCN-01 (0.1 $\mu$M), CGP 41251 (0.14 $\mu$M), GF 109203X (12 $\mu$M) and Ro 31-8220 (1.6 $\mu$M). Results are expressed as percentage difference from control cells and are the mean $\pm$s.d. of three experiments. Cell cycle distribution (in %) in control cells was as follows: 59.6$\pm$0.9 for $G_{0/1}$, 29.8$\pm$2.6 for S, 10.6$\pm$2.1 for $G_2$/M after 1 day; 63.3$\pm$2.0 for $G_{0/1}$, 25.5$\pm$0.9 for S and 10.8$\pm$1.1 for $G_2$/M after 2 days; 67.6$\pm$2.0 for $G_{0/1}$, 26.3$\pm$3.0 for S and 6.0$\pm$5.0 for $G_2$/M after 4 days. Asterisks indicate that values were significantly different from control cultures (*$P<0.05$; **$P<0.005$). For details of flow cytometric analysis see Materials and methods.

**Figure 4** Cycle distribution of A549 cells synchronised for 12 h with aphidicoline after 6, 12 and 24 h exposure to CGP 41251 (0.14 $\mu$M), GF 109203X (12 $\mu$M) and Ro 31-8220 (1.6 $\mu$M). Results are expressed as percentage difference from control (synchronised) cells and are the mean of two experiments. Cell cycle distribution (%) in control cells was as follows: 13.4 for $G_{0/1}$, 20.4 for S, 65.9 for $G_2$/M after 6 h; 81.5 for $G_{0/1}$, 9.0 for S and 9.5 for $G_2$/M after 12 h; 57.6 for $G_{0/1}$, 26.9 for S and 15.5 for $G_2$/M after 24 h. Results similar to those shown here were obtained with cells synchronised with nocodazole. For details of flow cytometric analysis see Materials and methods.

Cells were synchronised in M-phase with nocodazole (0.4 $\mu g\ ml^{-1}$), a reversible inhibitor of mitotic spindle formation, or in early S-phase with aphidicolin (2 mg $ml^{-1}$), a reversible DNA polymerase inhibitor, for 12 h. Subsequently, cells were washed, transferred to fresh medium with or without staurosporine analogue, fixed and analysed.

### *Cytotoxicity assay*

The cytotoxic potential of the compounds was established by measurement of release of lactate dehydrogenase (LDH) from cells grown in 24-well multidishes, using a LDH assay kit (Sigma). Cells were seeded at $7 \times 10^4$ per well and left for 24 h after which they were incubated for a further 24 h with drug at several concentrations in 0.5 ml medium containing 1% FCS. The amount of LDH released into the medium was measured. Intracellular LDH was liberated from cells adhering to the plate by lysis caused by treatment with 0.1% Triton X100 in PBS (0.5 ml) at 37°C for 30 min. LDH was determined using a Beckman DU 7500 spectrophometer. Enzyme leakage is expressed as percentage of total releasable LDH.

### Results

The compounds used in this study, staurosporine, UCN-01, CGP 41251, Ro 31-8220 and GF 109203X, have previously been shown to arrest the growth of A549 cells with $IC_{50}$ values ranging from 0.0007 $\mu$M (staurosporine) to 7.6 $\mu$M (GF 109203X) (Courage *et al.*, 1995). In those experiments cell growth was assessed after exposure to the drugs for 4 days (four doubling times), and the indolocarbazoles, staurosporine, UCN-01 and CGP 41251, were more potently cytostatic than the bisindolylmaleimides, Ro 31-8220 and GF 109203X. In the experiments described here, A549 cells were treated with staurosporine and its analogues for 1, 2 and 4 days and cell cycle distribution was studied. The drug concentrations used were equivalent to the $IC_{80}$ values

**Figure 5** Inhibition of growth of A549 cells after exposure for 2 (□) or 4 days (●) to staurosporine, UCN-01, CGP41251, Ro31-8220 and GF109203X. Results are the mean ±s.d. of three experiments, each conducted in duplicate. For experimental details see Materials and methods.

established previously for growth inhibition after exposure for 4 days (Courage *et al.*, 1995). Staurosporine and UCN-01 induced cell accumulation in $G_{0/1}$ (Figures 2 and 3) at all three time intervals investigated. CGP 41251 retarded cells weakly in $G_{0/1}$ and $G_2/M$ after 4 days, but had little or no effect on the cell cycle at the earlier time points. Both bisindolylmaleimides caused a slight increase in number of cells in $G_2/M$. In the case of GF 109203X this effect decreased with time, and on day 4 was superseded by weak retardation in $G_{0/1}$ (Figure 3).

Owing to the subtle nature of the effects observed, experiments were repeated using synchronised cells. Cells were exposed to nocodazole for 12 h after which they were released into medium containing staurosporine, UCN-01 or CGP 41251 and cell cycle distribution was examined after 6, 12 or 24 h. Similarly, cells were exposed to aphidicolin for 12 h after which they were released into medium containing CGP 41251, Ro 31-8220 or GF 109203X. Consistent with the results obtained with asynchronous cells, staurosporine and UCN-01 inhibited in $G_{0/1}$ (data not shown), whereas CGP 41251 had no effect on any phase of the cell cycle (Figure 4). Ro 31-8220-treated synchronised cells progressed with a delay through all stages of the cycle, and GF 109203X slowed cells in $G_2/M$ at the 12 and 24 h time points (Figure 4).

Cells treated with aphidicolin, nocodazole and the staurosporine analogues demonstrated an apparent shift of the DNA profile to the left compared with control cells. This phenomenon may be the consequence of a change in DNA stainability, which has also been observed after arrest of cells at the $G_1/S$ boundary with hydroxyurea (Z Darzynkiewicz, personal communication).

In the light of the time-dependent effects of the staurosporine analogues on the cell cycle, it seemed opportune to investigate how the cytostatic potency of the drugs is affected by duration of exposure time. Cells were incubated for 4 days with drug at three concentrations and cytostasis was compared with that observed after incubation for 2 days. Figure 5 shows that all five compounds were less potently growth inhibitory after 2 rather than 4 days, indicating time dependency. It has been suggested that inhibition of growth of some carcinoma cell types, including the MCF-7 cell line, by UCN-01 is irreversible on removal of the drug (Seynaeve *et al.*, 1993). In order to investigate whether this property is a general hallmark of staurosporine analogues, A549 cells were exposed to drugs either for 4 days or for 1 day, after which drug was removed and cells maintained in drug-free medium for 3 days. An 'irreversible block', such as that observed by Seynaeve *et al.* (1993) for UCN-01, did not occur in A549 cells. For all five compounds short-term treatment furnished weak but persistent cytostasis, which was less effective than that seen after continuous exposure for 4 days (Figure 6). In order to rule out an effect specific to these cells, the experiment was repeated using MCF-7 cells. Incubation of cells with UCN-01 for 1 or 2 days followed by maintenance in drug-free medium for 4 or 5 days, respectively, was markedly less growth inhibitory than continuous incubation with drug for 6 days (Figure 7), which is consistent with the results described above, but incompatible with those obtained by Seynaeve *et al.* (1993).

Finally, the concentrations of the staurosporine analogues which cause cytostasis were compared with those which elicit acute cytotoxicity, as measured by the cellular release of LDH. The rank order of cytotoxic potency was staurosporine > UCN-01 > Ro 31-8220 > CGP 41251 > GF 109203X (Figure 8). Staurosporine was the most potent inhibitor of cell growth and the most cytotoxic of the five compounds. Yet the difference between cytotoxic and cytostatic concentration, the latter adjudged by its $IC_{50}$ value of 0.65 nM (Courage *et al.*, 1995), was 15 000-fold. The analogous differences between cytotoxic and cytostatic concentrations for UCN-01 and Ro 31-8220 were 300- and 25-fold respectively. At 33 $\mu$M, the highest concentration used in the cytotoxicity assay without encountering solubility problems, neither CGP 41251 nor GF 109203X caused marked LDH release. On the assumption that cytotoxicity occurs at concentrations not far above this value, the difference between cytostatic and cytotoxic concentrations is probably ~400-fold for CGP 41251, but only ~4-fold for GF 109203X.

**Figure 6** Inhibition of growth of A549 cells caused by exposure to staurosporine, UCN-01, CGP41251, Ro31-8220 and GF 109203X after either 4 days (●) or 1 day followed by removal of drug and incubation in drug-free medium for 3 days (□). Results are the mean ±s.d. of three experiments, each conducted in duplicate. For experimental details see Materials and methods.

**Figure 7** Inhibition of growth of MCF-7 cells after exposure for 1 (□), 2 (●) or 6 days (▲) to UCN-01. Results are the mean ±s.d. of three experiments, each conducted in duplicate. For experimental details see Materials and methods.

**Figure 8** Cytotoxicity of staurosporine, UCN-01, CGP 41251, Ro 31-8220 and GF 109203X in A549 cells as judged by ability to elicit release of lactate dehydrogenase after exposure for 24 h. Results, which are expressed as percentage of total releasable enzyme in the cells, are the mean ±s.d. of three experiments, each conducted in duplicate. For experimental details see Materials and methods.

### Discussion

This study demonstrates that PKC-specific analogues of staurosporine with close structural similarity differ substantially in their effects on the cell cycle when applied at concentrations exerting growth arrest of comparable potency. Staurosporine and UCN-01 arrested cells in $G_{0/1}$. Ro 31-8220 slowed the transit of synchronised cells through all phases of the cell cycle, in asynchronous cells a small percentage of cells was maintained in $G_2M$. GF 109203X induced a potent $G_2$/M arrest in synchronised cells, but by day 4 this phenomenon had been replaced by a weak $G_{0/1}$ block. CGP 41251 had only an ephemeral effect on the cell cycle, which suggests that, at the concentration studied, it interferes with cell growth predominantly in a non-cycle-specific manner. CGP 41251 behaves differently from the other staurosporine analogues in other respects. For example, it is the only one of these agents which failed to cause translocation of PKC-ε from the cytosol to the membrane (Courage *et al.*, 1995). In the light of the close structural similarity between CGP 41251, staurosporine and UCN-01 (see Figure 1), these are intriguing pharmacological differences.

Cell cycle effects of the indolocarbazoles have been reported before, but in most of the published studies drug concentrations were considerably higher than those employed here. For example, staurosporine at 2.2 nM affected cells in $G_{0/1}$ in non-transformed cells, but not in transformed cells (Crissman *et al.*, 1991). At 50–100-fold higher concentrations it blocked both transformed and non-transformed cells in $G_2$/M (Crissman *et al.*, 1991; Abe *et al.*, 1991). Staurosporine at 5.8 nM delayed the progress of A431 cells through $G_2$/M transiently, and beyond 8 h it arrested cells in $G_{0/1}$ (Akinaga *et al.*, 1994). In contrast, at a 10-fold higher concentration it caused a profound $G_2$/M block. UCN-01 at 150 nM blocked MDA MB 468 cells from exiting $G_{0/1}$ and entering S-phase (Seynaeve *et al.*, 1993). This drug at 260 nM caused accumulation of A431 cells in $G_{0/1}$, but at 1.54 μM it delayed $G_2$/M progression transiently up to 12 h, followed by a $G_{0/1}$ block (Akinaga *et al.*, 1994). CGP 41251 at concentrations above those employed here has been shown to arrest cell cycle progression in $G_2$/M, at 0.5 and 1 μM in A549 cells and in NCI-H520 squamous carcinoma cells (Ikegami *et al.*, 1996); and at 10 μM in *ras*-transformed rat fibroblasts (Akinaga *et al.*, 1993). The results presented above, together with the relevant literature, are consistent with the notion that accumulation in $G_{0/1}$ might be important for the cytostasis exerted by the indolocarbazoles, staurosporine and UCN-01, at low concentrations. Effects of staurosporine analogues of the bisindolylmaleimide type on the cell cycle have, to our knowledge, hitherto not been described. Our results suggest that their primary cell cycle target is probably $G_2$/M. If inhibition of PKC activity was an important mechanistic determinant of the antiproliferation elicited by staurosporine analogues, one might surmise that agents of similar high specificity for PKC would exert similar effects on the cell cycle. This was not the case. Therefore, the results buttress the conclusion that inhibition of PKC *per se* is not the primary arbiter of the growth arrest caused by these compounds. This interpretration is consistent with a recent investigation in this laboratory which demonstrated that inhibition of PKC activity could not be directly related to cell growth arrest induced by these compounds (Courage *et al.*, 1995).

As PKC is not the prime determinant of the antiproliferative effect of these agents, what are the cellular signalling elements which are their major targets? Prime candidates are the cyclin-dependent kinases (cdks), which, in concert with cyclins, are vital components of the cell cycle machinery (Norbury and Nurse, 1992). There is some evidence supporting the notion that staurosporine and UCN-01 affect growth via cdks and/or cyclins. Staurosporine blocked the progression of human lymphocytes through $G_{0/1}$ between the cyclin D and cyclin E restriction points and markedly suppressed phytohaemagglutinin-stimulated cyclin E expression (Gong *et al.*, 1994). The staurosporine-induced $G_2$/M block in transformed cell lines has been shown to be due, at least in part, to inhibition of $p34^{cdc2}$ kinase (Gadbois *et al.*, 1992). UCN-01 at $G_{0/1}$-arresting concentrations inhibits cdks 2, 4 and 6 and decreases the amount of phosphorylated retinoblastoma susceptibility gene product (pRB) in A549 cells (Kawikami *et al.*, 1996). It remains to be elucidated whether CGP 41251 and the bisindolylmaleimides affect cyclins and/or cdks. The mechanism by which the indolocarbazoles exert higher growth-inhibitory potency than the bisindolylmaleimides involves perhaps differential abilities to interfere with cdks.

All five compounds inhibited cell growth more effectively after exposure for a longer rather than a shorter time period and their cytostatic ability was diminished by drug removal after 1 day. These observations indicate that to achieve therapeutic efficacy dose schedules might have to be chosen such that they yield significant drug levels over long periods of time. Intriguingly, UCN-01 has been shown to interrupt MCF-7 cell proliferation irreversibly necessitating only brief exposure (Seynaeve et al., 1993), which was not seen when the experiment was repeated in this laboratory. The difference between this result and that described by Seynaeve et al. (1993) may well be rooted in phenotype discrepancies between MCF-7 cells of different origin, a fact illustrated most poignantly by the discordant pattern of PKC isoenzyme expression in MCF-7 cells in different laboratories (Blobe et al., 1993; Stanwell et al., 1994).

One advantage of the treatment of cancer with modulators of signal transduction pathways might be the possibility that they exert cytostasis and not cytotoxicity, perhaps thus minimising undue toxicity to the host. It is noteworthy that, among the agents studied here, the indolocarbazoles displayed a greater difference between cytostatic and acute cytotoxic concentrations than the bisindolylmaleimides, insinuating the possibility of an analogous difference in safety margins.

In conclusion, the results presented here in concert with the work cited above suggest that (1) the mechanism via which bisindolylmaleimides arrest growth are different from those operative for indolocarbazoles; (2) PKC does not appear to play an important role in these mechanisms; (3) as far as the indolocarbazoles are concerned, cellular targets of CGP 41251 are probably different from those which mediate the growth effects of staurosporine and UCN-01. The mechanistic basis of the differences is unresolved, but it may well involve differential effects on cdks. Preclinical studies have not been published for Ro 31-8220 and GF 109203X, only for CGP 41251 and UCN-01 (Meyer et al., 1989; Akinaga et al., 1991), both of which are currently undergoing clinical evaluation as anti-cancer drugs. So the conclusions drawn here cannot be interpreted in the light of established pharmacological differences *in vivo*. The necessity of extended exposure time for efficacy and the significant margin between cytostatic and acute cytotoxic concentrations might be taken into consideration in forthcoming clinical trials of this type of antisignalling drug.

**Acknowledgements**

The work was supported by a generous grant from the Cancer Research Campaign of Great Britain (SP 2233). We thank Dr T Tamaoki (Kyowa Hakko Kogyo Co, Tokyo, Japan), Dr D Fabbro (Ciba Geigy, Basle, Switzerland) and Dr D Bradshaw (Roche Research Centre, Welwyn Garden City, UK) for samples of UCN-01, CGP 41251 and Ro 31-8220 respectively.

### References

ABE K, YOSHIDA M, USUI T, HORINOUCHI S AND BEPPU T. (1991). Highly synchronous culture of fibroblasts from G2 block caused by staurosporine, a potent inhibitor of protein kinases. *Exp. Cell Res.*, **192,** 122–127.

AKINAGA S, GOMI K, MORIMOTO M, TAMAOKI T AND OKABE M. (1991). Antitumor activity of UCN-01, a selective inhibitor of protein kinase C, in murine and human tumor models. *Cancer Res.*, **51,** 4888–4892.

AKINAGA S, NOMURA K, GOMI K AND OKABE M. (1993). Diverse effects of indolocarbazole compounds on the cell cycle progression of *ras*-transformed rat fibroblast cells. *J. Antibiotics*, **46,** 1767–1771.

AKINAGA S, NOMURA K, GOMI K AND OKABE M. (1994). Effect of UCN-01, a selective inhibitor of protein kinase C, on the cell-cycle distribution of human epidermoid carcinoma, A431 cells. *Cancer Chemother. Pharmacol.*, **33,** 273–280.

BLOBE GC, SACHS CW, KHAN WA, FABBRO D, STABEL S, WETSEL WC, OBEID LM, FINE RL AND HANNUN YA. (1993). Selective regulation of expression of protein kinase C (PKC) isoenzymes in multidrug-resistant MCF-7 cells. Functional significance of enhanced expression of PKCα. *J. Biol. Chem.*, **268,** 658–664.

COURAGE C, BUDWORTH J AND GESCHER A. (1995). Comparison of ability of protein kinase C inhibitors to arrest cell growth and to alter cellular protein kinase C localisation. *Br. J. Cancer*, **71,** 697–704.

CRISSMAN HA, GADBOIS DM, TOBEY RA AND BRADBURY EM. (1991). Transformed mammalian cells are deficiant in kinase-mediated control of progression through the G1 phase of the cell cycle. *Proc. Natl Acad. Sci. USA*, **88,** 7580–7584.

DAVIS PD, ELLIOTT L, HARRIS W, HURST SA, KEECH E, KUMAR H, LAWTON G, NIXON JS AND WILKINSON SE. (1992). Inhibitors of protein kinase C. 2. Substituted bisindolylmaleimides with improved potency and selectivity. *J. Med. Chem.*, **35,** 994–1001.

GADBOIS DM, HAMAGUCHI JR, SWANK RA AND BRADBURY EM. (1992). Staurosporine is a potent inhibitor of p34cdc2 and p34cdc2-like kinases. *Biochem. Biophys. Res. Commun.*, **184,** 80–85.

GONG J, TRAGANOS F AND DARZYNKIEWICZ. (1994). Staurosporine blocks cell cycle progression through G1 between the cyclin D and cyclin E restriction points. *Cancer Res.*, **54,** 3136–3139.

HENNIPMAN A, VAN OIRSCHOT BA, SMITS J, RIJKSEN G AND STAAL GE. (1989). Tyrosine kinase activity in breast cancer, benign breast disease, and normal breast tissue. *Cancer Res.*, **49,** 516–521.

IKEGAMI Y, YANO S AND NAKAO K. (1996). Antitumor effect of CGP 41251, a new selective protein kinase C inhibitor, on human non-small cell lung cancer cells. *Jpn. J. Pharmacol.*, **70,** 65–72.

KAWAKAMI K, FUTAMI H, TAKAHARA J AND YAMAGUCHI K. (1996). UCN-01, 7-hydroxyl-staurosporine, inhibits kinase activity of cyclin-dependent kinases and reduces the phosphorylation of the retinoblastoma susceptibility gene product in A549 human lung cancer cell line. *Biochem. Biophys. Res. Commun.*, **219,** 778–783.

MEYER T, REGENASS U, FABBRO D, ALTERI E, RÖSEL J, MÜLLER M, CARAVATTI G AND MATTER A. (1989). A derivative of staurosporine (CGP 41251) shows selectivity for protein kinase C inhibition and *in vitro* anti-proliferative as well as *in vivo* antitumour activity. *Int. J. Cancer*, **43,** 851–856.

NORBURY C AND NURSE P. (1992). Animal cell cycles and their controls. *Annu. Rev. Biochem.*, **61,** 441–470.

O'BRIAN CA AND WARD NE. (1989). Biology of the protein kinase C family. *Cancer Metast. Rev.*, **8,** 199–214.

POWIS G. (1992). Signalling targets for anticancer drug development. *Trends Pharmacol. Sci.*, **12,** 188–194.

SEYNAEVE CM, STETLER-STEVENSON M, SEBERS S, KAUR G, SAUSVILLE EA AND WORLAND PJ. (1993). Cell cycle arrest and growth inhibition by the protein kinase antagonist UCN-01 in human breast carcinoma cells. *Cancer Res.*, **53,** 2081–2086.

STANWELL C, GESCHER A, BRADSHAW TD AND PETTIT GR. (1994). The role of protein kinase C isoenzymes in the growth inhibition caused by bryostatin 1 in human A549 lung and MCF-7 breast carcinoma cells. *Int. J. Cancer*, **56,** 585–592.

TAMAOKI T AND NAKANO H. (1990). Potent and specific inhibitors of protein kinase C of microbial origin. *Biotechnology*, **8,** 732–735.

TOULLEC D, PIANETTI P, COSTE H, BELLEVERGUE P, GRAND-PERRET T, AJAKANE M, BAUDET V, BOISSIN P, BOURSIER E, LORIOLLE F, DUHAMEL L, CHARON D AND KIRILOVSKY J. (1991). The bisindolylmaleimide GF 109203X is a potent and selective inhibitor of protein kinase C. *J. Biol. Chem.*, **266,** 15771–15781.

TRINKS U, BUCHDUNGER E, FURET P, KUMP W, METT W, MEYER T, MÜLLER M, REGENASS U, RIHS G, LYDON N AND TRAXLER P. (1994). Dianilinophthalimides: Potent and selective, ATP-competitive inhibitors of the EGF receptor protein tyrosine kinase. *J. Med. Chem.*, **37,** 1015–1027.

UTZ I, HOFER S, REGENASS U, HILBE W, THALER W, GRUNICKE H AND HOFMANN J. (1994). The protein kinase C inhibitor CGP 41251, a staurosporine derivative with antitumor activity, reverses multidrug resistance. *Int. J. Cancer*, **57,** 104–110.